# Availability of post-hospital services supporting community reintegration for children with identified surgical need in Uganda

**DOI:** 10.1186/s12913-018-3510-2

**Published:** 2018-09-20

**Authors:** Emily R. Smith, Brittney J. van de Water, Anna Martin, Sarah Jean Barton, Jasmine Seider, Christopher Fitzgibbon, Mathama Malakha Bility, Nelia Ekeji, Joao Ricardo Nickenig Vissoci, Michael M. Haglund, Janet Prvu Bettger

**Affiliations:** 1 Duke Global Health Institute, Duke University, Durham, NC USA; 2 Department of Public Health, Robbins College of Health and Human Sciences, Baylor University, One Bear Place #97313, Waco, TX 76798 USA; 3 Department of Global Health and Social Medicine, Harvard Medical School, Boston, MA USA; 4 Duke University, Sanford School of Public Policy, Durham, NC USA; 5 Physical Therapy and Occupational Therapy, Duke University Medical Center, Durham, NC USA; 6 Duke University Divinity School, Durham, NC USA; 7 Department of Orthopedic Surgery, Division of Physical Therapy, Duke University, School of Medicine, Durham, NC USA; 8 Mechanical Engineering and Materials Science, Duke University, Durham, NC USA; 9 Triangle Insights Group, Durham, NC USA; 10 Duke University, Trinity College, Durham, NC USA; 11 Department of Surgery, Division of Emergency Medicine, Duke University, School of Medicine, Durham, NC USA; 12 Division of Global Neurosurgery and Neurology, Duke University, Durham, NC USA; 13 Department of Neurosurgery, Duke University, Durham, NC USA

**Keywords:** Pediatric, Community-reintegration, Surgical, Rehabilitation, Uganda

## Abstract

**Background:**

Community services and supports are essential for children transitioning home to recover from the hospital after surgery. This study assessed the availability and geographic capacity of rehabilitation, assistive devices, familial support, and school reintegration programs for school-aged children in Uganda with identified surgical need.

**Methods:**

This study assessed the geographic epidemiology and spatial analysis of resource availability in communities in Uganda. Participants were children with identified surgical need using the Surgeons OverSeas Assessment of Surgical need (SOSAS). Community-based resources available to children and adolescents after surgery in Uganda were identified using publicly available data sources and searching for resources through consultation with in-country collaborators We sought resources available in all geographic regions for a variety of services.

**Results:**

Of 1082 individuals surveyed aged 5 to 14 yearsr, 6.2% had identified surgical needs. Pediatric surgical conditions were most prevalent in the Northern and Central regions of Uganda. Of the 151 community-based services identified, availability was greatest in the Central region and least in the Northern region, regardless of type. Assuming 30% of children with surgical needs will need services, a maximum of 50.1% of these children would have access to the needed services in the extensive capacity estimates, while only 10.0% would have access in the minimal capacity estimates. The capacity varied dramatically by region with the Northern region having much lower capacity in all scenarios as compared to the Central, Eastern, or Western regions.

**Conclusions:**

Our study found that beyond the city of Kampala in the Central region, community-based services were severely lacking for school-aged children in Uganda. Increased pediatric surgical capacity to additional hospitals in Uganda will need to be met with increased availability and access to community-based services to support recovery and community re-integration.

## Background

Disability is a major health concern. An estimated 15% of the world’s population is living with a disability and 80% of persons with disabilities live in developing countries [1]. Children share a disproportionate burden. One-third of the world’s population of people with disabilities is children, and 65% of disabilities are estimated to be preventable [2]. Children are most susceptible to disability due to their increased dependency on others [3–5]. The major causes of disability among children in low- and middle-income countries (LMICs) include war, infectious disease, malnutrition, dangerous living and working conditions, injuries caused by trauma or accidents (predominantly road), and congenital conditions [1, 6]. Many of these conditions require surgical intervention [7, 8], and previous research has found the lifetime prevalence of surgical conditions for children ranges from 10 to 85% in LMICs, with the most common conditions being trauma-related, congenital deformities, and masses [8–10]. In Uganda, the site of the current study, the prevalence of pediatric surgical conditions was 17% with 65% of these needs being unmet. Surgical intervention, although cost-effective and beneficial in LMICs, can be delayed or not provided due to limited access to trained surgeons and adequately equipped hospitals [11]. However, even if treated, many surgical conditions carry the risk of life-long disability and have limited value without proper follow-up [8].

Once discharged from the hospital, children may require assistive devices, rehabilitation, and other specialized social and educational supports to address activity limitations and reduce barriers to participation [12–14]. Unfortunately, these provisions are not readily available in many LMICs, and are especially limited for children [15]. For example, children require physically smaller medical devices such as wheelchairs, braces, and gastrostomy tubes, supplies that are often limited even for adults. Social and educational needs of children with disabilities can also be difficult to address following hospital discharge; according to UNESCO, 98% of children with disabilities in low-income countries do not attend school. [16] Without appropriate services and supports, children with postoperative disabilities face further health complications, isolation, and stigmatization [17, 18]. For children with disabilities to be integrated and valued community members, it is critical that services be made available and accessible.

The objective of this study was to determine the availability and geographic distribution of community-based rehabilitation, assistive devices, familial support and school reintegration programs for school-aged children in Uganda with identified surgical need. In addition, the availability of the community-based rehabilitation programs was compared with the pediatric surgical need by country region. Assessing the gap between children in need of surgery and the availability of post-operative services in low-income countries is an important foundation from which to build referral strategies, partnerships, and policies to support children and their families.

## Methods

### Setting

The study setting of Uganda was chosen based on our previous work on pediatric surgical conditions in the country [10]. Uganda has a population of approximately 38 million people, with 49% of inhabitants aged 14 years or younger [19]. As a nation classified by the World Bank as low-income, Uganda is 83% rural with 19.7% of its population living below the poverty line [19, 20]. Of the total health expenditure, Uganda allots 7.2% of its gross domestic product (GDP) to health, with nearly half of health-related expenditures as out-of-pocket payments [19, 21]. The healthcare system of Uganda has two national referral hospitals located in the capital city of Kampala (one for adult general medicine, pediatrics, and surgery [Mulago National Referral Hospital], and the other for psychiatry [Butabika National Referral Hospital]). The Mulago National Referral Hospital provides the highest level of pediatric surgical care in the country. Additionally, there are 14 regional referral hospitals and 139 district hospitals throughout the country, which can provide basic emergency surgical procedures [22].

### Population

In 2014, the Surgeons OverSeas Assessment of Surgical need (SOSAS) questionnaire was administered to individuals within households surveyed across 105 enumeration areas (EAs) in Uganda using a two-stage, cluster-randomized sampling design, described elsewhere [23]. In short, the households were randomly selected based on geographic sub-regions to collect data based on proportional-to-size methodology to obtain a national representative sample. The SOSAS study surveyed 4248 individuals in 2315 households of which 1082 (24.4%) were considered to be school-aged children between 5 and 14 years of age [23]. In Uganda, primary school begins at age 6 and is 7 years in length [24]. Each identified surgical condition was rated by two or more surgeons and medical/surgical trainees as surgically-treatable and non- surgically treatable conditions. Among the surgically-treatable conditions, each case was coded by surgeons or surgical trainees as treated or untreated based on whether the patient received appropriate surgical care [9, 25, 26].

For this study, we included children with identified surgical needs related to injuries, acquired deformities, and congenital deformities as these children would likely need services and supports post-operatively [27–32]. Our selected sub-sample of *N* = 1082 comprised 43.9% of the identified surgical needs among children from birth to 14 years of age.

### Community-based services and supports

Community-based resources available to children and adolescents after surgery in Uganda were first identified using publicly available data sources. These sources included government reports, directories of disability organizations such as those compiled by the Uganda Society for Disabled Children (USDC) [33], UNICEF’s research study on children with disabilities living in Uganda [34], and the Community Based Rehabilitation Africa Network (AfriCAN) [35]. We attempted to include all services, regardless if they were public or private and type of funding stream. Our inventory of services and supports was further developed with input from local leaders and community organizers in Uganda between June 2016 – June 2017. Eight researchers worked in pairs to identify resources within four domains of community-based, postoperative services: 1) rehabilitation services (ES, JS); 2) assistive devices and technologies (SB, CF); 3) school reintegration programs (AM, MB); and 4) social or family support services (SB, NE).

The scope of each domain was guided by the service types included in the environmental domain of the International Classification of Functioning, Disability and Health-Children and Youth [36]. Rehabilitation services were further defined to include physical therapy, occupational therapy, community-based rehabilitation, and advocacy groups (those specifically related to disability rights, disability sensitization, and groups promoting access to therapeutic services). Assistive devices included organizations that provide medical and assistive devices for children with disabilities. Social support and family services included organizations that provide social support services to families (food security, work training, funding, education, rehabilitation resources), parent and caregiver advocacy meetings (at local, country, and continent level meetings), parent, family and caregiver support groups, as well as pediatric medical homes with children living with post-surgical disabilities and/or conditions. The domain for school reintegration included organizations that provide advocacy and assistance related to accessing educational institutions (i.e. financial assistance), or actual educational services for children with disabilities post-surgery. School reentry programs were identified as a day school, residential school, learning center, as well as support, advocacy, or facilitating group. Organizations that offered services solely for non-surgical conditions were excluded. Services exclusively available to children with vision or hearing impairment and services specific to people living with HIV/AIDS were excluded as these services were not inclusive of the primary surgical needs in our study population. The team contacted organizations via telephone or email to collect information not available from public data sources. The geographic coordinates for each identified resource were obtained using Google maps and recorded in our database of community-based services and supports.

### Availability of services

The community-based services were mapped by region using quantum geographic information system (QGIS) version 2.8 [37]. *Availability* of services is described as the total number of services available for each of the four domains (rehabilitation, assistive devices, social and family support, school re-entry) at the country and regional levels. Uganda is divided into four administrative regions: Central includes the nation’s capital of Kampala, Eastern borders Kenya and includes Mbale, Western includes the mountainous region and the city of Mbarara, and the Northern region extends across the country sharing borders with Kenya, the South Sudan and the Democratic Republic of the Congo [38].

### Capacity

Capacity was defined as the availability of services within each region for post-operative surgical care for children. Using the availability of services per region, we compared three capacity estimatess for post-operative, community-based services for children with surgical needs. The base population consisted of the number of school-aged children with surgical needs, estimated from the number of children within each region and the percentage of school-aged children with surgical needs from previous reports [9, 39]. Among these children, the three scenarios included estimates of 10%, 20%, and 30% of children with surgical needs estimated to need services post-surgery. The range of percentages were based on the number of children in four low-income countries with injuries, acquired deformities, and congenital deformities as these children would likely need services and supports post-operatively [9, 26]. Within each scenario, we examined capacity at the domain level using the number of children geographically located within each region based on a previous geospatial analysis, detailed elsewhere [25]. For each of the three scenarios of need we determined whether those children would have minimal, moderate, or extensive capacity to rehabilitation, assistive devices, social and family supports, and school re-integration services. Minimal capacity was defined as each organization serving 100 children, moderate capacity was defined as each organization serving 250 children, and extensive capacity was defined as each organization serving 500 children.

### Analysis

The prevalence of surgical conditions for school-age children in Uganda was estimated based on the SOSAS survey, by the Ugandan region. Demographic characteristics among children who reported a surgical condition were stratified and compared by region, using a weighted model. Household and individual cases were weighted using design weights for each enumeration area, household-level and individual-level response rates, and known population counts of gender and age groupings from the Uganda Census 2014 data. Post-surgical rehabilitative community-based resources were linked to the SOSAS survey data by region. We analyzed the data using SAS 9.4 (SAS Institute Inc., Cary, NC, USA) and stored the data in Microsoft Excel 2010 (Microsoft Corp, Redmond, WA, USA).

This study was approved by the Makerere University School of Medicine Research and Ethics Committee and Duke University Health System IRB.

## Results

Among 1082 children surveyed the median age was 9.5 years (interquartile range 7.1, 12.0) and there were slightly more females than males in each region, with the exception of the Eastern region (Table 1). More than 80% lived in rural areas. The majority of children reported being healthy within the past 12 months prior to the time of the interview, with no difference by region. The presence of surgical needs was higher in the Northern and Central regions (8.8% and 7.6% respectively) than in the Western and Eastern regions (4.9% and 4.4% respectively).

**Table 1:** Demographic characteristics, presence of unmet surgical conditions in school-aged children interviewed in SOSAS, stratified by regions of Uganda

		Uganda Regions				
	Total (*n* = 1082)	Central (*n* = 235)	Eastern (*n* = 280)	Western (*n* = 237)	Northern (*n* = 330)	
Demographic characteristics	n (%) Median (IQR)	n (%) Median (IQR)	n (%) Median (IQR)	n (%) Median (IQR)	n (%) Median (IQR)	*p*-value
Age (years)						
Median (IQR)	9.5 (7.1, 12.0)	9.6 (7.1, 12.2)	9.1 (7.0, 11.8)	9.9 (7.5, 12.6)	9.3 (7.2, 11.7)	–
Gender						
Male	518 (48.5)	109 (45.9)	151 (55.3)	145 (44.8)	113 (48.2)	0.04
Female	564 (51.5)	126 (54.1)	129 (44.8)	185 (55.2)	124 (51.8)	
Village type						
Rural	880 (81.5)	198 (82.0)	221 (81.3)	264 (80.2)	197 (82.6)	0.13
Urban	202 (18.5)	37 (18.0)	59 (18.7)	66 (19.8)	40 (17.4)	
Healthy in the past 12 months						
Yes	974 (89.7)	216 (90.2)	254 (91.5)	296 (89.6)	208 (87.3)	0.31
No	108 (10.2)	19 (9.8)	26 (8.5)	34 (10.5)	29 (12.7)	
Surgical needs	%	%	%	%	%	*p*-value
Injuries, acquired/ congenital deformity	6.2	7.6	4.4	4.9	8.8	0.45

Interquartile Range (IQR) = 25th and 75th percentiles

**Fig. 1:**
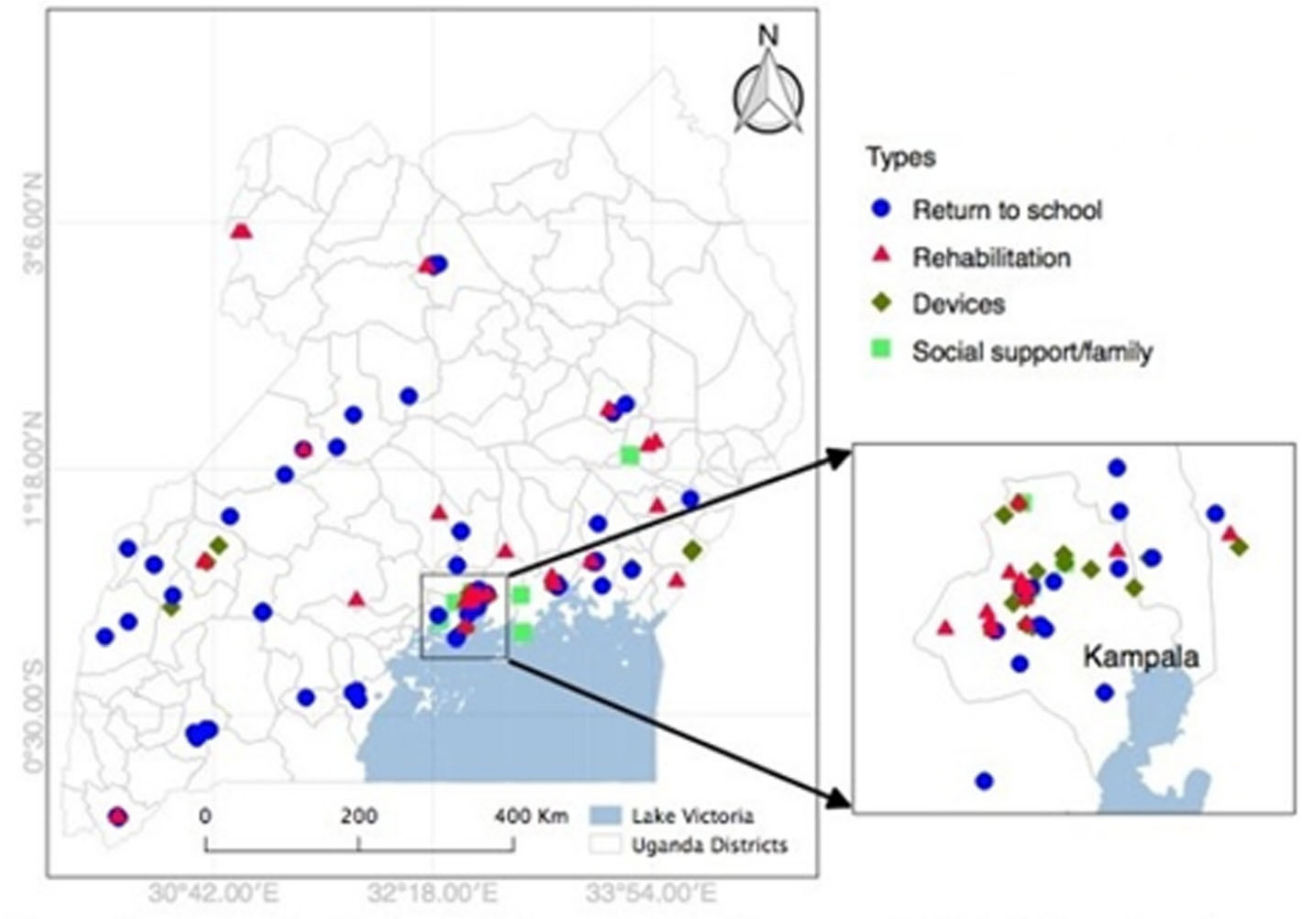
Community-based Services and Supports by Domain for Children with Surgical need in Uganda and Kampala

**Fig. 2:**
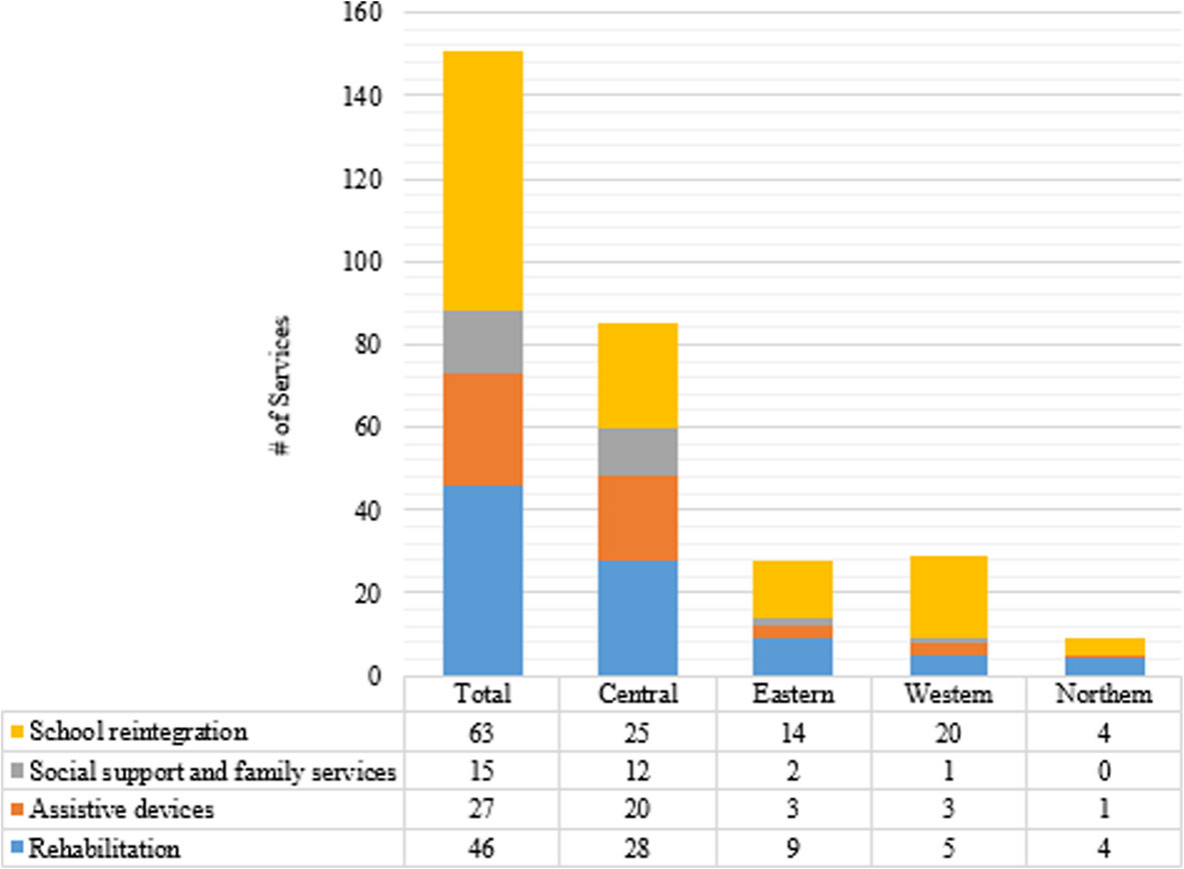
Presence of Community-based Services, Stratified by Region of Uganda

There were 151 community-based services located throughout the country, with more than half in the Central region (56.3%) and fewest in the Northern region (6.0%), regardless of type of service (Fig. 1). Of the 151 services across all regions, 63 (41.7%) were for school reintegration, 46 (30.5%) were for rehabilitation services, 27 (17.9%) were for assistive devices, and 15 (9.9%) were for social support and family services (Fig. 2). The Eastern, Western and Northern regions had 2, 1, and 0, social support and family services identified, respectively.

Table 2 describes three scenarios of need and three projections for levels of capacity to community-based services (minimum, moderate, extensive) in Uganda. Assuming 30% of children with surgical needs will need post-surgical services, a maximum of 50.1% of these children would have access to the needed services in the extensive capacity estimates, while only 10.0% would have access in the minimal capacity estimates. The capacity varied dramatically by region with the Northern region having 15.0% capacity in the extensive scenario as compared to the Central, Eastern, or Western regions (83.7%, 50.8%, and 26.3%, respectively).

**Table 2:** Capacity estimatess of services for school-aged children pediatric surgical needs

		Uganda Regions			
	Total	Central	Eastern	Western	Northern
Base population					
School-aged children (number)	8,098,000	2,226,950	2,089,284	2,089284	1,692,482
Children with surgical needs* (%)	6.2	7.6	4.4	8.8	5.9
Children with surgical needs of (number)	502,076	169,248	91,928	183,856	99,856
Capacity estimatess for children with surgical needs					
Scenario 1: 30% require services	150,622	50,774	27,578	55,157	29,956
Minimal capacity (%)	10.0	16.7	10.2	5.3	3.0
Moderate capacity (%)	25.1	41.9	25.4	13.1	7.5
Extensive capacity (%)	50.1	83.7	50.8	26.3	15.0
Scenario 2: 20% require services	100,415	33,849	18,385	36,771	19,971
Minimal capacity (%)	15.0	25.1	15.2	7.9	4.5
Moderate capacity (%)	37.6	62.8	38.1	19.7	11.3
Extensive capacity (%)	75.2	100.0	76.1	39.4	22.5
Scenario 3: 10% require services	50,207	16,924	9192	18,385	9985
Minimal capacity (%)	30.1	50.2	30.5	15.8	9.0
Moderate capacity (%)	75.2	125.6	76.1	39.4	22.5
Extensive capacity (%)	100.0	100.0	100.0	78.9	45.1

Minimal capacity was defined as each organization (*n* = 151) serving 100 children, moderate capacity was defined as each organization serving 250 children, and extensive capacity was defined as each organization serving 500 children

*injuries, acquired/congenital deformities

In scenario 2, if 20% of children with surgical needs would require services, a maximum of 75.2% of children would have access in the extensive capacity estimates, while only 15.0% would have access in the minimal capacity estimates. The Northern region had the lowest capacity for all three scenarios than the other regions reaching a maximum of 39.4% in the maximum capacity estimates.

The final scenario assumed 10% of children with surgical needs require post-surgical services. Approximately 100% of these children would have access to needed services at the most extensive capacity estimates, while 30% would have capacity in the minimal scenario. The capacity reached 100% for the Central region in the moderate scenario and the capacity reached 100% in the Eastern region in the extensive scenario. The capacity never reached 100% for the Western and Northern regions.

## Discussion

Our study found that beyond the city of Kampala and the region surrounding the national referral hospital, access to post-operative, community-based services was severely lacking for school-aged children in Uganda. Although the need for community-based post-surgical services for children was greatest in the Northern region, availability and access were most limited in this area of the country. Increased pediatric surgical capacity among hospitals in Uganda will need to be met with increased availability and access to community-based services.

Access to post-surgical community-based services and supports for children in LMICs, particularly sub-Saharan Africa, is often limited due to sparse and unpredictable funding, limited training for personnel, decreased societal prioritization of pediatric post-surgical care, and little awareness of unique needs for children despite the large burden of disability [9, 40, 41]. This study illustrates a dearth of post-hospital resources outside of Kampala to ensure successful rehabilitation and increased participation in community, home, and school contexts. Among adults, lack of postoperative care and services immediately after surgery increases the likelihood of complications including infection, hemorrhages, and excessive pain [42]. Children also suffer from complications when services are not available in the immediate postoperative phase of care [17]. This study illustrates the need for a comprehensive strategy in support of surgical care and recovery across the continuum of care and community reintegration.

UNICEF estimates that roughly 20% of children with disabilities have access to healthcare services and only 10% to rehabilitation services in Uganda [34]. The prevalence of children with disability has, unfortunately, increased over time [1]. The present study shows that the majority of community-based services available are distributed within the Central and Eastern regions of Uganda, resulting in unmet need in the Western and Northern regions. The high rates of unmet needs are also seen in other LMICs. In Rwanda, a national survey of musculoskeletal impairments found 2.6% of children were impaired, 80,000 needed physical therapy, and 10,000 needed assistive devices [43]. An assessment of orthopedic surgical capacity in East, Central, and Southern Africa found 36% of district hospitals in rural areas had rehabilitation units with very few hospitals having a trained rehabilitation specialists [44]. One way to expand capacity is to strengthen the link between hospitals and rehabilitation services through direct referral systems and educational interventions while in the hospital to the family caregivers. However, a key barrier on this linkage is the inadequate numbers of skilled healthcare workers for pediatric surgery and rehabilitation services throughout Uganda. In LMICs, both adult and pediatric surgery numbers are affected by lack of surgeons, anesthesiologists, and supporting staff [45]. In Uganda, there are only 5 pediatric surgeons and one pediatric anesthetist in the country with 18 million children under the age of 18, translating to a ratio of 0.03 providers per 100,000 children. In comparison, in the United States there were approximately 3 pediatric surgeons per 100,000 in 2009 [46], and that proportion has likely increased over the past 7 years. Skilled workers and training facilities for rehabilitation professionals are even more sparse in LMICs [47]. Thus, future recommendations should include linkages from hospital to the home after a child has a surgical procedure but should also include increased in training of rehabilitation professionals.

Many of the existing clinics and residential programs have limited patient capacity. Residential schools for children with disabilities integrate several rehabilitations services, but also offer outpatient therapy for children in the community as an effective strategy to expand their capacity. Physical rehabilitation is essential to promote improved functioning, independence, and community reintegration for children with disability. These outcomes ultimately assist children to develop active, contributory roles in society and enhance quality of life. Expanding capacity for clinics and rehabilitation homes to reach children with unmet need in communities could be a cost-effective approach to improve long-term health and societal outcomes.

Most of the community-based resources are from non-governmental and/or donor-based agencies with funding streams from Danish, Norweigan, and American development agencies that have been engaged in the disability movement in the country for the pasts few decades. Uganda has a strong advocacy presence in the Ugandan Parliament for adults with disabilities with representation from the five regions in the country [47]. However, there are few national and government-run programs for children with disabilities in the country. With the push towards universal health capacity in LMICs, the incorporation of rehabilitative services after a healthcare procedure should be considered in close collaboration with in-country stakeholders, programs, and governmental advocacy groups.

Despite the lack of community-based rehabilitation and transitional programs for children leaving the hospital after surgery, excellent models of pediatric rehabilitative care in sub-Saharan Africa exist. [48, 49] In conducting this study, we learned about one organization, Comprehensive Rehabilitation Services in Uganda (CoRSU), that has a holistic model of care providing different types of therapy, services from psychologists and social workers, educational services, and specialty medical care for children in need of orthopedic and plastic surgery [50]. On a national level, policies, delivery systems, and healthcare laws could be reformed to incorporate national rehabilitation plans, particularly in the era of universal health coverage. In addition, funding mechanisms to increase human resources for rehabilitative services and expand service delivery, through decentralizing care, would also improve services for children.

This study has several limitations. We recognize care for children goes beyond the home and the transitional period immediately after hospitalization. Rehabilitation and transitional care should be holistic, thus, the chosen domains were not mutually exclusive. With some organizations listed in two or three separate domains, these services could appear inflated, thus increasing the estimation of independent service providers in a certain catchment area. Methods for finding sites were not error proof. Internet searches, government sponsored reports, and independent or research related program reports were employed to locate organizations. Given two individuals checked each domain, we feel as though we conducted a thorough search; however, new organizations are initiated regularly, and many organizations go out of business, do not staff a ‘brick and mortar’ office in Uganda, or have closed. Additionally, ascertaining the number of providers/technicians staffed, patients treated, beds available, or devices provided annually for each site was difficult; among many organizations, this information is not tracked or not publicly available. It is also possible that some organizations were not identified because they were not specific to pediatrics but may have the capacity and resources to provide services for children.

## Conclusion

This study identified community-based rehabilitation, assistive device, education, and family social support services for school-aged children with post-surgical needs in all regions of Uganda; however, access was severely geographically limited. Future research will aim to build relationships between existing services and hospitals caring for children with surgical needs, and to identify areas of greatest need for capacity building. Working with Ugandan organizations and stakeholders to gain insight into the daily burden and needs of children after surgery will provide more context for future research. Ultimately, community-based services supporting hospitalized school-aged children in their post-hospital transition to their home community should be available to all in order to improve functioning and encourage participation to the fullest extent in all situations [3, 4, 36, 51, 52]. Preventing or minimizing disability through age- and culturally-appropriate community-based services is paramount in providing children opportunities to fully participate in all activities after surgical intervention.

## Abbreviations

AfriCAN: Community Based Rehabilitation Africa Network
CoRSU: Comprehensive Rehabilitation Services in Uganda
EAs: Enumeration areas
GDP: Gross domestic product
LMICs: Low- and middle-income countries
QGIS: Quantum geographic information system
SOSAS: Surgeons OverSeas Assessment of Surgical need
UNCRPD: United Nations Convention on the Rights of Persons with Disabilities
USDC: Uganda Society for Disabled Children

## References

[1] Organization WH (2011). World report on disability.

[2] Cameron DL, Nixon S, Parnes P, Pidsadny M (2005). Children with disabilities in low-income countries. Paediatr Child Health.

[3] Erikson EH. Childhood and society. 2d, rev. and enl. ed. New York U6 - ctx_ver=Z39.88–2004&ctx_enc=info%3Aofi%2Fenc%3AUTF-8&rfr_id=info%3Asid%2Fsummon.serialssolutions.com&rft_val_fmt=info%3Aofi%2Ffmt%3Akev%3Amtx%3Abook&rft.genre=book&rft.title=childhood+and+society&rft.au=Erikson%2C+Erik+H.+1902–1994.+%28Erik+Homburger%29&rft.date=1964-01-01&rft.pub=Norton&rft.externalDocID=b12412326&paramdict=en-US U7 - Book: Norton; 1964.

[4] Bronfenbrenner U (1979). Contexts of child rearing: problems and prospects. Am Psychol.

[5] Bronfenbrenner U (1981). The ecology of human development: experiments by nature and design.

[6] Abdelgadir JMD, Smith ERP, Punchak MM, Vissoci JRP, Staton CMD, Muhindo AMD (2017). Epidemiology and characteristics of neurosurgical conditions at Mbarara regional referral hospital. World Neurosurg.

[7] Bickler SW, Rode H (2002). Surgical services for children in developing countries. Bull World Health Organ.

[8] Ozgediz D, Poenaru D (2012). The burden of pediatric surgical conditions in low and middle income countries: a call to action. J Pediatr Surg.

[9] Butler EK, Tran TM, Fuller AT, Brammell A, Vissoci JR, de Andrade L (2016). Quantifying the pediatric surgical need in Uganda: results of a nationwide cross-sectional, household survey. Pediatr Surg Int.

[10] Fuller AT, Butler EK, Tran TM, Makumbi F, Luboga S, Muhumza C (2015). Surgeons OverSeas Assessment of Surgical Need (SOSAS) Uganda: Update for Household Survey. World J Surgery.

[11] Saxton AT, Poenaru D, Ozgediz D, Ameh EA, Farmer D, Smith ER (2016). Economic analysis of Children’s surgical Care in low- and Middle-Income Countries: a systematic review and analysis. PLoS One.

[12] Peranich L, Reynolds KB, O'Brien S, Bosch J, Cranfill T (2010). The roles of occupational therapy, physical therapy, and speech/language pathology in primary care. Int J Nurs Pract.

[13] Trovato MMD, Kim HMD, Moberg-Wolff EMD, Murphy NMD, Kim CTMDP (2010). Pediatric rehabilitation: 4. Prescribing assistive technology to promote community integration. PM R.

[14] O'Sullivan SB, Schmitz TJ, Fulk GD (2014). Physical rehabilitation.

[15] Rose JMD, Weiser TGMD, Hider PF, Wilson LF, Gruen RLP, Bickler SWP (2015). Estimated need for surgery worldwide based on prevalence of diseases: a modelling strategy for the WHO Global Health estimate. Lancet Glob Health.

[16] United Nations Educational SaCO (2009). Toward Inclusive Education for Children with Disabilities: A Guideline.

[17] Penny N, Zulianello R, Dreise M, Steenbeek M (2007). Community-based rehabilitation and orthopaedic surgery for children with motor impairment in an African context. Disabil Rehabil.

[18] Kishiki EBA, Shirima SM, Lewallen SMD, Courtright PD (2009). Improving postoperative follow-up of children receiving surgery for congenital or developmental cataracts in Africa. J AAPOS.

[19] Agency CI (2017). The World Factbook.

[20] Bank W (2016). The Uganda poverty assessment report 2016.

[21] Kagumire R (2009). Public health insurance in Uganda still only a dream. Can Med Assoc J.

[22] Health UMo. Hospitals 2018 [Available from: http://health.go.ug/affiliated-institutions/hospitals.

[23] Fuller AT, Haglund MM, Lim S, Mukasa J, Muhumuza M, Kiryabwire J (2016). Pediatric neurosurgical outcomes following a neurosurgery health system intervention at Mulago Hospital in Uganda. World Neurosurg.

[24] FHI360. Uganda: National Education Profile (2014). FHI360: Education Policy and Data Center.

[25] Smith ER, Vissoci JRN, Rocha TAH, Tran TM, Fuller AT, Butler EK (2017). Geospatial analysis of unmet pediatric surgical need in Uganda. J Pediatr Surg.

[26] Butler EK, Tran TM, Nagarajan N, Canner J, Fuller AT, Kushner A (2017). Epidemiology of pediatric surgical needs in low-income countries. PLoS One.

[27] Calisti A, Belay K, Mazzoni G, Fiocca G, Retrosi G, Olivieri C (2011). Promoting major pediatric surgical Care in a low-Income Country: a 4-year experience in Eritrea. World J Surg.

[28] Toobaie A, Emil S, Ozgediz D, Krishnaswami S, Poenaru D (2017). Pediatric surgical capacity in Africa: current status and future needs. J Pediatr Surg.

[29] Ameh E, Bickler S, Lakhoo K, Nwomeh B, Poenaru D. Organization GH, editor. Pediatric Surgery: A Comprehensive Text for Africa. Seattle. WA2011

[30] Abimanyi-Ochom J, Mannan H (2014). Uganda’s disability journey: progress and challenges. Afr J Disabil.

[31] United Nations. Convention on the rights of persons with disabilities. United Nations; 2015. https://www.un.org/development/desa/disabilities/convention-on-the-rights-of-persons-with-disabilities.html.

[32] Uganda MoHRo. Ministry of Health: Republic of Uganda 2017. Available from: http://www.health.go.ug/.

[33] Uganda Society for Disabled Children (no date). Disability Organizations in Uganda: A Directory of National and District Organizations of and for Persons with Disabilities.

[34] Riche N, Anyimuzala J. Research study on children with disabilities living In Uganda. UNICEF; 2014.

[35] (CAN) CBRCAN. CBR Directory for Africa 2017 [Available from: http://afri-can.org/cbr-directory-for-africa/

[36] Organization WH (2007). Internal classification of functioning - children and youth version.

[37] QD T. QGIS geographic information system: Open source geospatial foundation project 2016. Available from: https://qgis.org/.

[38] Uganda Bureau of Statistics (2012). Uganda demographic and health survey 2011.

[39] Uganda Bureau of Statistics. National Population and Housing Census 2014 - Main Report. Kampala: Uganda Bureau of Statistics; 2014. p. 77–86.

[40] Ademuyiwa AO, Usang UE, Oluwadiya KS, Ogunlana DI, Glover-Addy H, Bode CO (2012). Pediatric trauma in sub-Saharan Africa: challenges in overcoming the scourge. J emergencies, trauma, and shock.

[41] Adeoye A, Seeley J, Hartley S (2011). Developing a tool for evaluating community-based rehabilitation in Uganda. Disabil Rehabil.

[42] Hobson B, et al. (n.d.). “An Introduction to Postoperative Complications,” University College London. https://www.ucl.ac.uk/anaesthesia/StudentsandTrainees/Intro_to_postop_Complications. Accessed Nov 2016.

[43] Atijosan O, Simms V, Kuper H, Rischewski D, Lavy C (2009). The orthopaedic needs of children in Rwanda: results from a national survey and orthopaedic service implications. J Pediatr Orthop.

[44] Chokotho L, Jacobsen KH, Burgess D, Labib M, Le G, Peter N (2016). A review of existing trauma and musculoskeletal impairment (TMSI) care capacity in east, central, and southern Africa. Injury.

[45] Meara JG, Leather AJ, Hagander L, Alkire BC, Alonso N, Ameh EA (2015). Global surgery 2030: evidence and solutions for achieving health, welfare, and economic development. Lancet.

[46] Poley S, Ricketts T, Belsky D, Gaul K (2009). Pediatric surgeons: subspecialists increasing fastor than generalists.

[47] http://www.who.int/disabilities/world_report/2011/chapter4.pdf.

[48] Mweshi M, Amosun S, Ngoma M, Nkandu E (2011). Managing children with spina bifida in sub-Saharan Africa: the Zambian experience?. Med J Zambia.

[49] Organization WH (2004). Review of disability issues and rehabilitation services in 29 Africa countries.

[50] CORSU. Comprehensive rehabilitation services in Uganda 2017 [Available from: http://corsu-uganda.org.

[51] Holmbeck GN (2002). A developmental perspective on adolescent health and illness: an introduction to the special issues. J Pediatr Psychol.

[52] Kaufman M (2006). Role of adolescent development in the transition process. Prog Transplant.

